# MOF Synthesis Prediction Enabled by Automatic Data Mining and Machine Learning**

**DOI:** 10.1002/anie.202200242

**Published:** 2022-03-10

**Authors:** Yi Luo, Saientan Bag, Orysia Zaremba, Adrian Cierpka, Jacopo Andreo, Stefan Wuttke, Pascal Friederich, Manuel Tsotsalas

**Affiliations:** ^1^ Institute of Functional Interfaces Karlsruhe Institute of Technology Hermann-von-Helmholtz-Platz 1 76344 Eggenstein-Leopoldshafen Germany; ^2^ Institute of Nanotechnology Karlsruhe Institute of Technology Hermann-von-Helmholtz-Platz 1 76344 Eggenstein-Leopoldshafen Germany; ^3^ Basque Center for Materials, Applications & Nanostructures Edif. Martina Casiano, Pl. 3 Parque Científico UPV/EHU Barrio Sarriena 48940 Leioa Bizkaia Spain; ^4^ Institute of Theoretical Informatics Karlsruhe Institute of Technology Am Fasanengarten 5 76131 Karlsruhe Germany; ^5^ Ikerbasque Basque Foundation for Science Bilbao 48013 Spain; ^6^ Institute of Organic Chemistry Karlsruhe Institute of Technology Kaiserstrasse 12 76131 Karlsruhe Germany

**Keywords:** Data Mining, Machine Learning, Metal–Organic Frameworks, Microporous Materials, Synthesis Prediction

## Abstract

Despite rapid progress in the field of metal–organic frameworks (MOFs), the potential of using machine learning (ML) methods to predict MOF synthesis parameters is still untapped. Here, we show how ML can be used for rationalization and acceleration of the MOF discovery process by directly predicting the synthesis conditions of a MOF based on its crystal structure. Our approach is based on: i) establishing the first MOF synthesis database via automatic extraction of synthesis parameters from the literature, ii) training and optimizing ML models by employing the MOF database, and iii) predicting the synthesis conditions for new MOF structures. The ML models, even at an initial stage, exhibit a good prediction performance, outperforming human expert predictions, obtained through a synthesis survey. The automated synthesis prediction is available via a web‐tool on https://mof‐synthesis.aimat.science.

## Graphical abstract

An approach to rationalize and accelerate MOF discovery by directly predicting the synthesis conditions of a MOF based on its crystal structure is reported. The prediction is based on machine learning models, trained on the SynMOF database, constructed via automatic data mining of synthesis parameters from the literature. The model outperforms human expert predictions according to a synthesis survey.

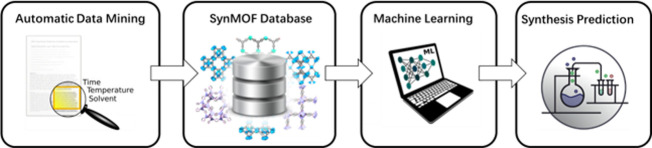

Metal–organic framework (MOF) chemistry has flourished through the creation of a vast chemical space where more than 100 000 MOFs have been discovered.^[1]^ The number is increasing rapidly with a wide and continuously expanding variety of structural types, building units, linkage chemistry, and functional groups.^[2]^ In fact, the chemical space of possible MOF structures exceeds millions of structures, which makes it impossible to fully explore experimentally.^[3]^ Simulation and machine learning (ML) have evolved as important tools for guiding researchers to computationally identify regions of interest.[^3a^, ^4^] However, in order to synthesize the novel MOF structures, the researchers still have to rely on their experience, employing a trial‐and‐error approach (Figure 1). This is a very challenging process that is highly time‐consuming, labor‐intensive, and requires a lot of resources. Therefore, the search for an efficient way to find the optimal MOF synthesis conditions represents the current bottleneck in speeding up MOF exploration.

**Figure 1:**
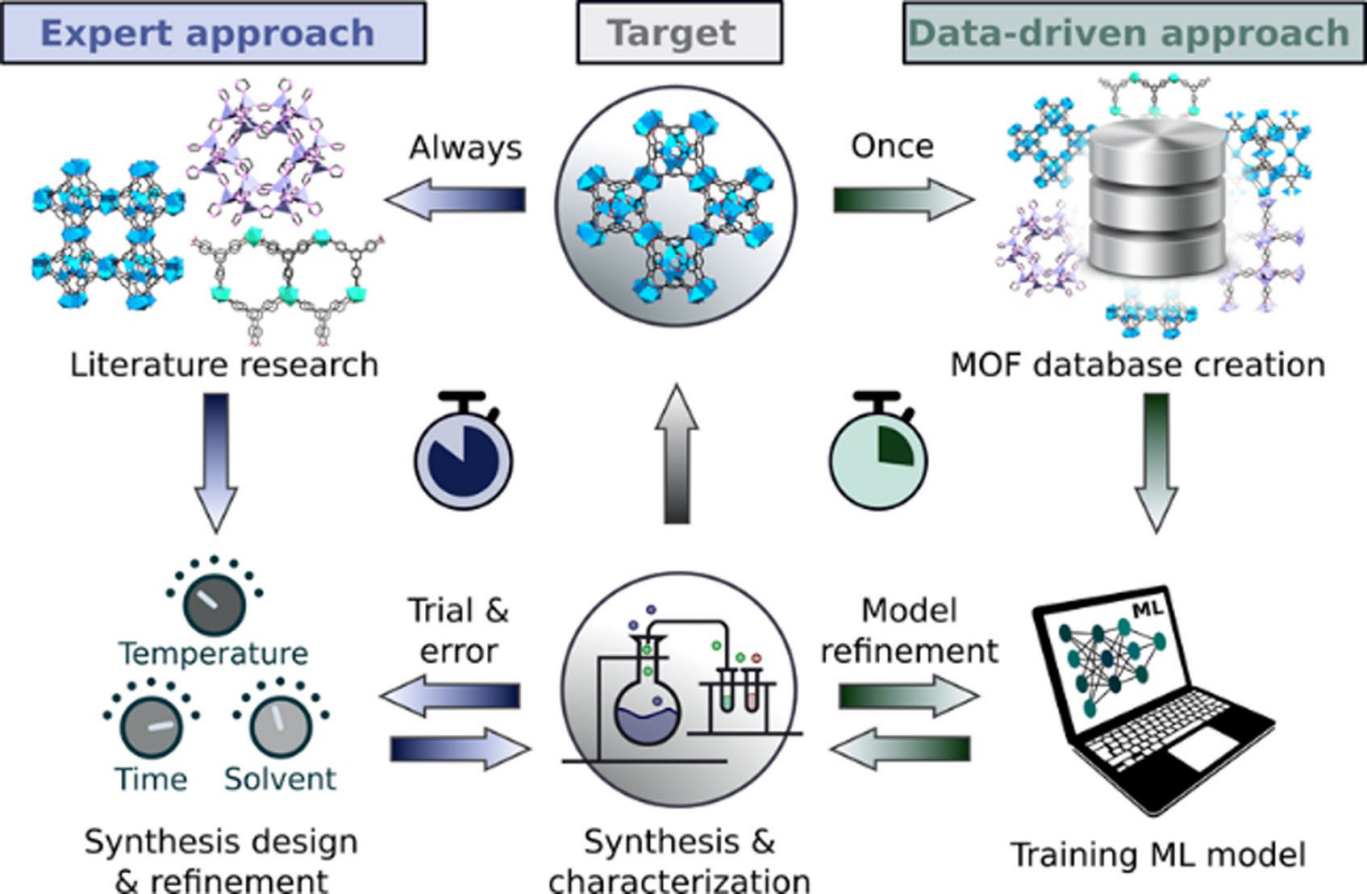
A new approach to MOF synthesis. The conventional approach (left loop) of new MOF synthesis is based on a time‐consuming trial‐and‐error approach, in which a target MOF structure is compared with reported MOFs from literature to find similar synthesis conditions and experimentally refine them. A data‐driven approach (right loop), where a ML model is trained on a library of automatically extracted literature data, to then suggest synthesis conditions in a data‐driven MOF discovery cycle. Updating the ML model based on new experiments leads to continuous improvement of the predictions.

The development of ML methods to predict the synthesis parameters for a desired MOF crystal structure based on scientific literature is a challenging but promising approach that will advance and accelerate chemical synthesis. Over the last years, ML methods have rapidly evolved, solving complex problems that involve highly nonlinear or massively combinatorial processes that conventional approaches fail to answer.^[5]^ Up till now, ML approaches have been successfully applied to address challenges in organic and inorganic synthesis.[^4a^, ^6^] In the case of MOF synthesis, only recently, ML was used to optimize synthesis parameters for HKUST‐1 and to determine the importance of the different parameters by analysing a set of partially failed experiments, in other words, “capture the chemical intuition” that can help to speed up the synthesis of similar MOF systems.^[7]^ However, the inverse synthesis design of MOFs, i.e. the automated prediction of suitable synthesis conditions for a targeted MOF structure (e.g. designed in silico) remains an unsolved challenge.

This work represents a first step towards predicting synthesis conditions for an arbitrary MOF. We show a complete ML workflow for the inverse synthesis design of MOFs (going from crystal structure to synthesis conditions), 1) starting from automated data mining from scientific literature on MOF synthesis conditions and their structural information, 2) setting up and training of ML models, and 3) prediction of synthesis conditions for new MOF structures and comparison with human experts’ predictions. Our approach marks the starting point for the transition from a trial‐and‐error approach that is based on experience and heuristics, towards an inverse synthesis design approach in the MOF synthesis, ultimately enabling fully autonomous MOF discovery in automated labs.^[8]^

To create a dataset with MOF synthesis parameters and structural information, we took advantage of the fact that well‐curated MOF structural databases already exist (e.g. the Computation‐Ready Experimental Metal–Organic Framework database CoRE MOF^[9]^ and the Cambridge Structural Database CSD^[10]^), in which MOF structural information and the corresponding publications with successful synthesis protocols are stored. The manual extraction of synthesis procedures from scientific literature is a time‐consuming task, requiring the work of many experts. Alternatively, automatic data extraction to convert experimental procedures into a set of the desired synthesis parameters by employing natural language processing (NLP) techniques is a highly efficient and promising approach that we expect to be continuously improved in the upcoming years.^[11]^

In this study, we developed an automatic process to extract information on MOF synthesis for all publicly available MOF structures in the CoRE MOF database (Supporting Information Section 2.1). The six relevant parameters that were extracted are metal source(s), linker(s), solvent(s), additive, synthesis time, and temperature (Figure 2). To achieve this, we initially classified literature paragraphs, employing a decision tree with a string search method, to identify the synthesis paragraph related to each MOF structure (Supporting Information Section 2.2). After the synthesis paragraphs were determined, we employed the ChemicalTagger software, which focuses on the experimental part of a scientific text, recognizing significant words within the sentences, and annotating phrases inside the paragraph.^[12]^ In an effort to increase the tagging accuracy, we slightly modified the synthesis paragraphs, accounting for MOF‐domain specific descriptions (Supporting Information Section 2.3). To evaluate the accuracy of the automatically extracted SynMOF‐A database, we additionally generated manually corrected versions—the SynMOF‐M and SynMOF‐ME databases that are discussed in Supporting Information Section 2.4.

**Figure 2:**
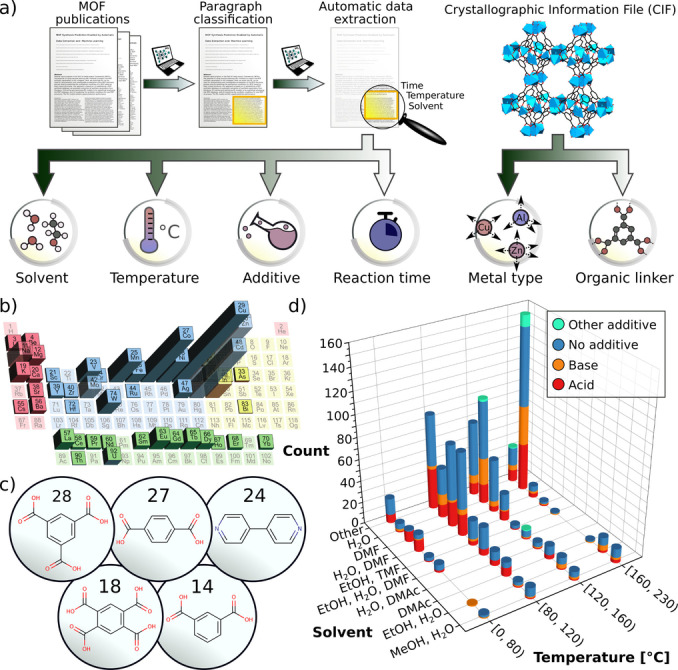
SynMOF database. a) Data mining pipeline and content of the SynMOF database; b) the statistics on the most common metal source and c) structures and occurrences of the most common linkers in the SynMOF database; d) 3D graph exhibiting correlation between solvent type, additive, and temperature.

Alongside retrieving synthesis information from the MOF literature, we used the crystallographic information files (CIFs) from MOF databases to automatically extract the structural information of the linker and the oxidation state of the metal center.^[13]^ Ultimately, we combined the extracted synthesis details (i.e. metal source, linkers, temperature, synthesis time, solvents, and additives) from the publications and information of the linker and the metal source from the CIF into the SynMOF database (Figure 2). Our central assumption in this work is that the established SynMOF database can be used to train ML models to facilitate the discovery of similarity patterns in the synthesis conditions to reach the final goal of predicting synthesis protocols for new MOF structures.

Apart from the detailed information on MOF synthesis conditions, our SynMOF database, currently consisting of 983 MOF structures, provides the statistical data on the metal source and organic components (Figure 2b, c). It contains 46 different metals with most common oxidation states ranging from +1 to +3. As expected, most MOF structures are composed of transition metals, with copper and zinc comprising almost 50 % of all metal types. Among the diverse organic molecules, the most commonly employed linkers for MOF synthesis are multidentate carboxylic acids (i.e., benzene‐1,3,5‐tricarboxylic acid, benzene‐1,4‐dicarboxylic acid, and benzene‐1,2,4,5‐tetracarboxylic acid) followed by N‐containing bases (i.e. pyridine, triazole, and tetrazole).

In search of obvious patterns, we analysed the most common solvents used during MOF synthesis with respect to different temperature regimes and additives (Figure 2d). At temperatures ranging from 80 °C to 160 °C, DMF and water, as well as their mixtures with other solvents are the most commonly used solvents. Synthesis at temperatures above 160 °C is predominantly carried out in water as a single solvent. Besides, the majority of MOF synthesis reactions at high temperatures (above 120 °C) are performed without additives, while at temperatures below 80 °C, the addition of acidic additives dominates. Beyond such relatively simple patterns, we expected more correlations to be hidden in the data (Supporting Information Section 2.5), which we exploit using ML approaches.

Employing the data stored in the SynMOF database, we trained multiple ML models to predict synthesis conditions of a diverse set of MOFs unseen during training. The input representation of the MOF structures is of crucial importance for the ML models’ performance.^[14]^ In this study, we used two types of representations as an input for the ML models training: One based on molecular fingerprints of the linkers, extended with encodings of the metal type and its oxidation state (Figure 3a, Supporting Information Section 3.1), and the recently developed MOF representation by Kulik and co‐workers (Supporting Information Section 3.2).^[15]^ It is to be noted that the MOF field is still expanding, and an increasing amount of new structures and corresponding synthesis parameters will be available over time that can be used for training and refinement of ML models to achieve the highest possible performance. In this case, representation learning methods such as graph neural networks will then likely become more accurate than models relying on hand‐crafted feature representations.^[16]^

**Figure 3:**
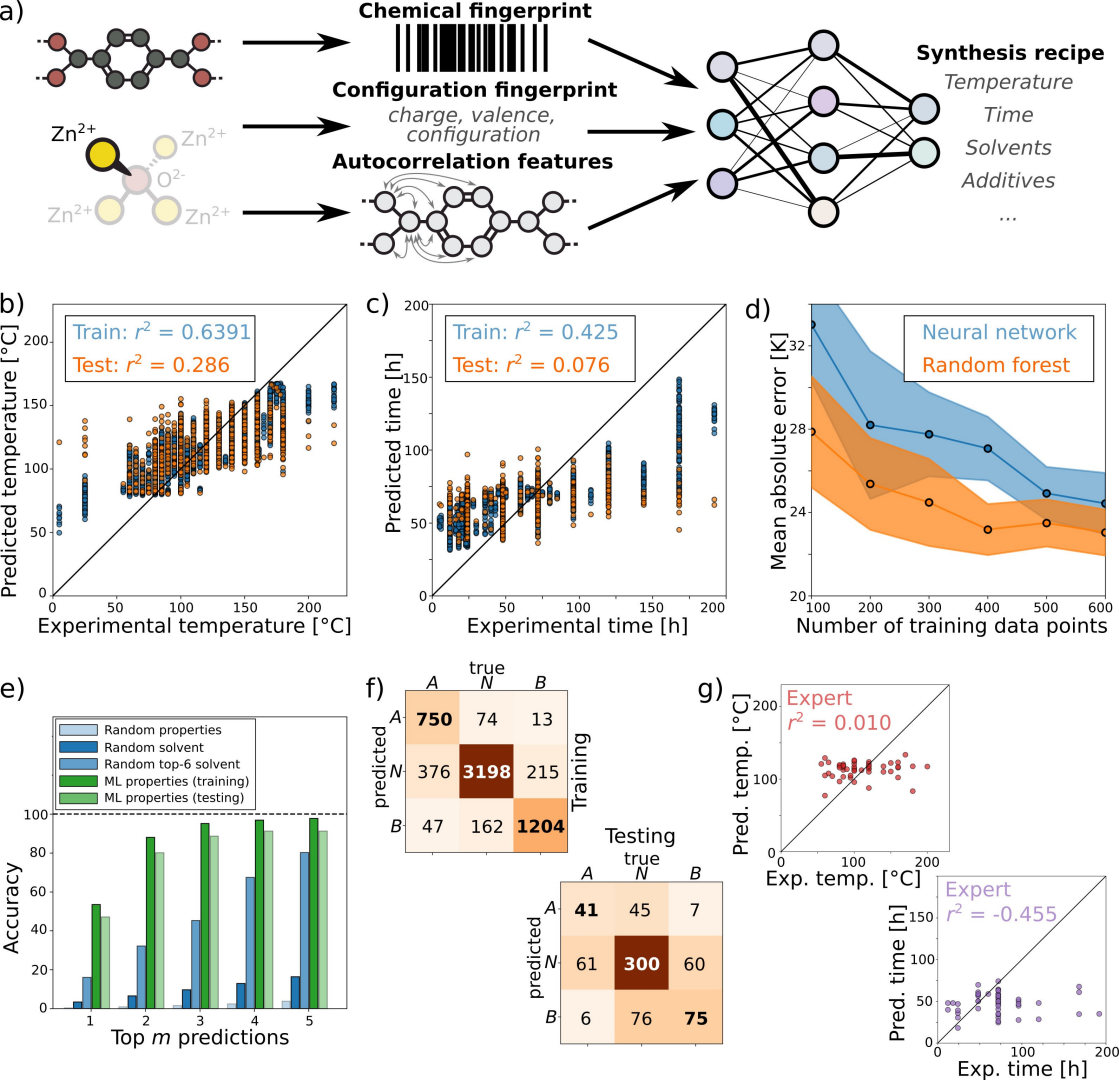
Machine learning models trained on the SynMOF‐A database. a) ML workflow, including fingerprint representation of the linkers and the feature representation of the metal type and oxidation state; b) and c) comparison of ML predictions of temperature and time for training and test sets with the initial data extracted from literature; d) learning curve of temperature predictions, i.e. mean absolute error as a function of the training set size, for neural network and random forest regression models; e) ML solvent prediction accuracy for a subset of single‐solvent MOFs, compared to different methods of random predictions; f) training and test set performance of additive classification where A, B, and N correspond to acid, base, and no additive respectively and g) average of eleven human expert predictions of temperature and time for 50 MOFs to evaluate the complexity of the problem.

The prediction of synthesis time and temperature was achieved via regression models, such as random forests or neural networks (Supporting Information Sections 3.3, 3.4, 3.5). To predict discrete synthesis parameters, such as solvent and additives, classification models could be, in principle, used. However, for multiple reasons this turns out to be impractical: There is a wide variety of possible solvents and additives reported in literature, leading to a large number of categories, and, in turn, strongly imbalanced datasets. Furthermore, the properties of solvents can be very similar, making them interchangeable in synthesis, which leads to ambiguous solutions. In practice, also combinations of various solvents are required for successful MOF synthesis. Therefore, we developed a ML model which predicts solvent properties, such as partition coefficients, boiling point (Supporting Information Section 3.6), rather than the specific solvent. A nearest neighbor search in solvent property space yields lists of possible solvents that have properties similar to those predicted by the ML model. In this way, new solvents can be incorporated easily, and even solvents occurring only once in literature can be used to train the model. In the case of additives, we found that the main parameter that distinguishes different additives is their acidity/basicity strength. Thus, we split the dataset into three groups (acidic, basic, or no additive) and used a classification model for additive prediction.

The results of our trained ML models are shown in Figure 3b–f. Reproducibly positive correlation coefficients *r* ^2^ on unseen test datasets show that the ML models can identify meaningful and predictive relations between the target MOF structure and the required synthesis conditions, in particular temperature and time (Figure 3b, c). Given the amount of data that we have currently extracted from literature, we find that the random forest models have the highest performance across all predicted parameters. However, neural networks learn to make better predictions with growing dataset sizes faster (see learning curves in Figure 3d) and even exploit correlations between different synthesis parameters (e.g., solvent and temperature) rather than predicting them separately. Hence, we expect that more complex models will outperform random forests in the near future.

To evaluate ML‐based solvent prediction, we focused on a subset of MOFs which are synthesized using only one solvent. We compared the accuracy of the top 6 ML predictions with multiple random baseline methods (Figure 3e), including selection of a random solvent out of all solvents as well as out of the six most frequent solvents that are used in 96 % of the single‐solvent SynMOF database. We found that the ML model outperforms the random selection, in particular for the top 1–3 solvent predictions, where the ML model reaches an accuracy of>90 %. In the case of additive predictions (Figure 3f), the task of the ML model is to classify required additives as acidic, basic, and no additive. While performing well on the training set, the generalization to unseen test data suffers from an imbalanced dataset (most database entries do not use an additive). We use balance correcting weights of the training data points, leading to predictions that distinguish very well between synthesis procedures involving basic and acidic additives. However, the differentiation between acidic and no additive or basic and no additive is less pronounced. One of the reasons might be related to the hidden variables such as type and function of additives: Some of them (inorganic acids and bases) have only the role of pH regulation, while others (organic acids and bases) are also involved in modulation of the MOF growth. Besides, concentration and strength of additives are additional important parameters, influencing the role of additives. A larger amount of training data in the future will enable refinement of the additive representation and improvement of our ML model, thus opening new prospects in synthesis condition prediction.

We note that the prediction of (MOF) synthesis conditions is an ill‐defined task, as there is not one true answer, but a whole range of conditions that lead to a successful synthesis. The data published in literature is very heterogeneous, as only some reactions are optimized for yield or other targets. Also, depending on the particular MOF, there might be wider or narrower windows for nearly‐optimal conditions. Therefore, in contrast to other machine learning applications, it is unlikely that even a perfect model will approach an *r* ^2^ score of 1. To put the ML performance into perspective, we performed tests with 11 human MOF synthesis experts. We developed an online quiz based on 50 MOFs randomly selected from the SynMOF database which will be publicly available. The participants were given the 3D structures of MOFs, chemical structures of the linkers and information on the metal ion, and asked to estimate synthesis conditions such as temperature, time, solvents, and additives without any help from literature or other external sources (Supporting Information Section 3.7). After each MOF synthesis prediction, we also asked the participants to estimate how certain they are in the answer. The correlation coefficients *r* ^2^ between the experts’ temperature and time predictions and the reported synthesis conditions are close to zero, even after averaging over 11 estimates by different researchers (Figure 3g) and after sorting only by predictions with high certainty. This rather surprising result shows that even small correlations learned and exploited by the ML model will help to estimate better synthesis conditions. In summary, we showed that the ML models are able to learn generalized patterns and correlations in the SynMOF database, which exceed the experts’ general intuition, and thus, could be used to identify good first guesses for experimental synthesis attempts of new MOFs.

We have developed a web site to predict of MOF synthesis conditions with our models, available via https://mof‐synthesis.aimat.science/. By using the web‐tool, the user can upload their own MOF CIFs. The web‐tool then predicts the synthesis conditions of the corresponding MOFs, including synthesis temperature, time, solvent, and additive (acid, base, or no additive).

In conclusion, the lack of machine‐readable and curated MOF‐synthesis data up till now hindered the development of digital ML tools for predicting MOF synthesis conditions. Here, we established a SynMOF database by automatic data extraction via NLP methods that provides synthesis conditions and structural information for more than 900 MOFs, and trained ML models based on these data to identify patterns in MOF synthesis. We expect that the created SynMOF database will boost the NLP research within the MOF community, while our ML synthesis prediction platform will be the new gold standard for data‐driven MOF discovery. Even at an initial stage, our ML models outperformed MOF experts′ synthesis prediction, underlying both the complexity behind the synthesis process and a pressing need in developing digital predictive tools. Our automated on‐demand synthesis prediction will considerably accelerate the discovery of new MOFs and serve as a valuable tool for the MOF community and beyond.

## Conflict of interest

The authors declare no conflict of interest.

## Supporting information

As a service to our authors and readers, this journal provides supporting information supplied by the authors. Such materials are peer reviewed and may be re‐organized for online delivery, but are not copy‐edited or typeset. Technical support issues arising from supporting information (other than missing files) should be addressed to the authors.

Supporting Information

## Data Availability

The databases SynMOF‐A, SynMOF‐M and SynMOF‐ME, the codes for the synthesis parameter extraction, for ML training and prediction, and the expert survey are available free of charge on https://github.com/Tsotsalas‐Group/MOF_Literature_Extraction and https://github.com/aimat‐lab/MOF_Synthesis_Prediction.
