# Supplementary material for: MOF Synthesis Prediction Enabled by Automatic Data Mining and Machine Learning
Source: Angew Chem Int Ed Engl. 2022 Mar 10;61(19):e202200242. doi: 10.1002/anie.202200242 (PMC9310626; doi:10.1002/anie.202200242)
Supplement: Supplementary file 1 — Supporting Information [file ANIE-61-0-s001.pdf]

Angewandte Chemie
Eine Zeitschrift der Gesellschaft Deutscher Chemiker

Supporting Information

MOF Synthesis Prediction Enabled by Automatic Data Mining and Machine Learning

Y. Luo, S. Bag, O. Zaremba, A. Cierpka, J. Andreo, S. Wuttke, P. Friederich, M. Tsotsalas**

Supplementary Information

Contents

1. Overview of the method of machine learning workflow 2
1.1 Accessibility of data, models, and web-based prediction tool 2
1.2 The general description of data mining and ML models 2
2. Overview of the automated SynMOF database generation 4
2.1. Data mining workflow from MOF literature 4
2.2. Decision tree algorithm for paragraph classification. 6
2.3. MOF synthesis conditions extraction 6
2.4. SynMOF databases 8
2.5. SynMOF database visualization 10
3. Details on the machine learning models 18
3.1. Fingerprint-based featurization 18
3.2. Features from Kulik and co-workers 19
3.3. Random forest (RF) regression models 19
3.4. Random forest (RF) classification models 19
3.5. Neural Network (NN) regression models 20
3.6. Solvent prediction 21
3.7. Details on expert survey 23
3.8. Demo for the web-tool 25
References 25

1. Overview of the method of machine learning workflow

Our ML workflow of the inverse synthetic design of MOFs (going from crystal structure to synthetic conditions) consists of three steps: (1) data mining from MOF scientific literature; (2) training ML models; (3) ML prediction and evaluation.

1.1 Accessibility of data, models, and web-based prediction tool

The data mining step and the ML models can be found on https://github.com/Tsotsalas-Group/MOF_Literature_Extraction and https://github.com/aimat-lab/MOF_Synthesis_Prediction. A web site (https://mof-synthesis.aimat.science/) has been launched according to this method to predict MOF synthesis condition based on crystallographic information file (CIF) of MOF structure.

1.2 The general description of data mining and ML models

In the data mining step, we extracted the synthesis conditions from MOF publications using different NLP techniques. To select synthesis paragraphs, we developed a decision tree algorithm based on a keyword list selected from 100 MOF synthesis papers. To analyse the synthesis paragraph and identify information about chemical entities, experimental steps, and corresponding conditions associated with those steps, we applied the ChemicalTagger software. When precursors, solvents and additives, as well as solvothermal synthesis conditions were extracted, we compared the metal element from the automatically formed synthesis protocol to the CoRE MOF database to eliminate mismatched conditions. The results of this fully automated data extraction are collected in the SynMOF-A database. We also evaluated the consistency of our automatically extracted database SynMOF-A with a manually extracted database SynMOF-M of the same MOF structures.

In the machine learning step, we developed a code to extract the MOF linker from the CIF. The RDKit library was further used to evaluate the molecular fingerprint of the extracted linker. The MOF metal nodes were represented by their full electronic configuration. The molecular fingerprint of the linker and the full electronic configuration of the metal node, accounting for its oxidation state, were combined to form the input of the ML model. This input representation was compared to the MOF representation developed by Kulik and co-workers, relying on autocorrelation features of the metal cores and the linkers. The output of the ML model was the MOF synthesis conditions, namely temperature, synthesis time, solvent properties and additive type. Depending on the specific synthesis conditions, we evaluated several regression models, in particular random forest regression and neural networks. The scikit-learn library in Python was used for the implementation of the ML models. 70% of the full dataset was used to train the ML model, while the remaining data was used to test the model. In the case of solvent property prediction, we limited the data to MOFs with single-solvent synthesis. To quantify the accuracy of the trained ML model, we calculated the mean absolute error and the correlation coefficient r2 of the training and test dataset for the regression tasks. The accuracy of the ML model for the classification tasks were quantified by calculating the normalized confusion matrix.

Finally, to rationalize the prediction accuracy and to estimate the complexity of the task, we compared the ML predictions to MOF experts' synthesis prediction.

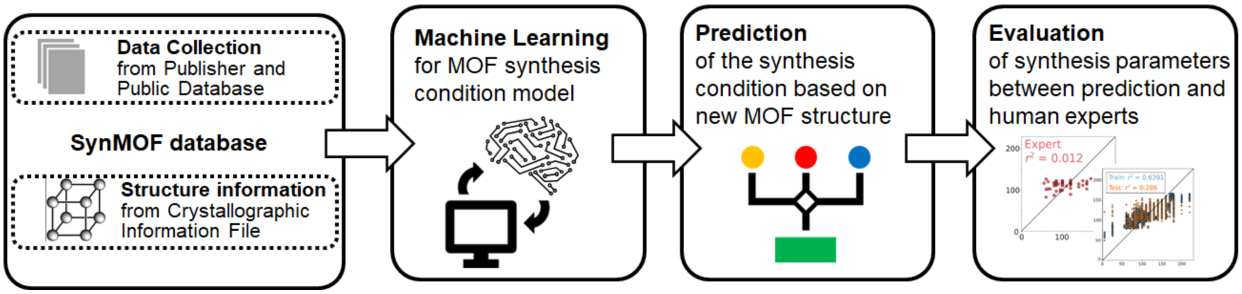

Figure S1. *Workflow of the system: (1) setup of a SynMOF database, containing the crystal structures and corresponding synthesis conditions via automatic extraction of synthesis conditions from scientific literature and structural information from crystal structure databases; (2) feature engineering for the MOF crystal structures and training of different ML approaches; (3) evaluation of the different ML approaches to predict the synthesis conditions for new MOFs.*

2. Overview of the automated SynMOF database generation

The content of our SynMOF database is based on the CoRE MOF database. From CoRE MOF, the CIF and corresponding reference papers were automatically downloaded and combined with additional information from the CSD. This information was analyzed by different natural language processing (NLP) techniques. **Figure S2** provides an overview of the SynMOF database generation.

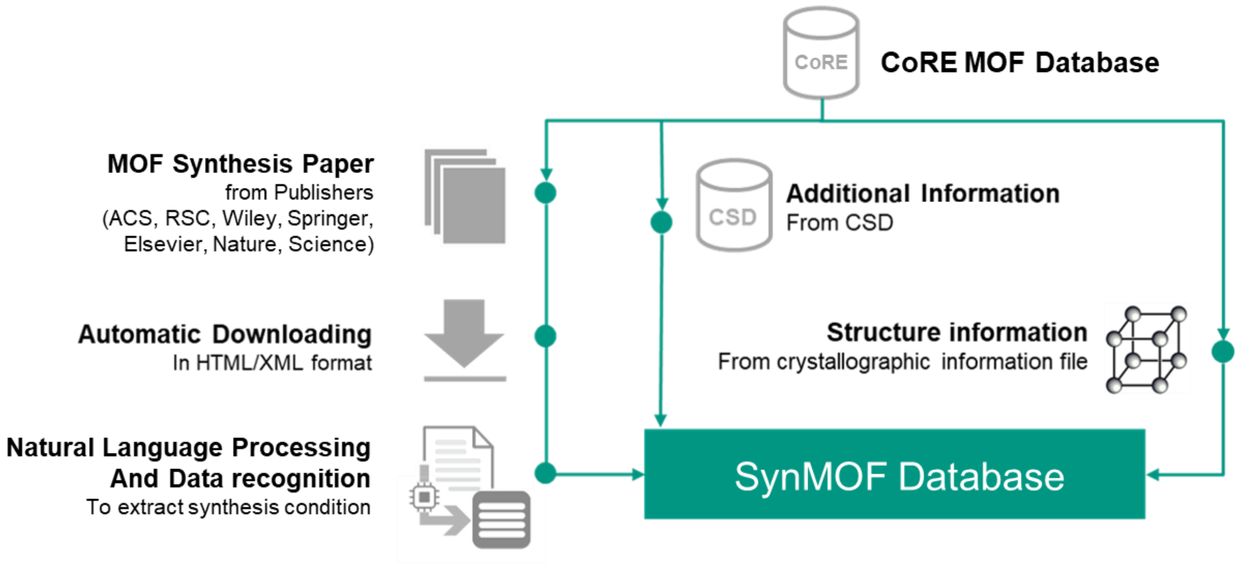

Figure S2. *Overview of the SynMOF database generation.*

2.1. Data mining workflow from MOF literature

From the 12,020 publicly available MOF structures in the CoRE MOF database, we selected 11,475 structures containing Cambridge Structural Database (CSD) identifiers in order to collect their deposition number and publication information using the CSD Python application programming interface (API)[1]. Based on the publication information, we created a web-scraping tool with Puppeteer (https://pptr.dev/) that allowed us to download 6099 journal articles, containing 10989 structures from the following publishers:

Springer, Wiley-VCH, Elsevier, the Royal Society of Chemistry, and the American Chemical Society. The downloaded papers included both the main manuscript and the supporting information. In order to preserve the files integrity, we stored all the data as original files (HTML/XML format) and developed an index search program. In the next step, the information was analyzed by different NLP techniques to identify the correct synthesis procedure paragraphs for each structure in the MOF publications and automatically extract the correct synthesis procedure. **Figure S3** shows the data mining workflow from literature developed in this study.

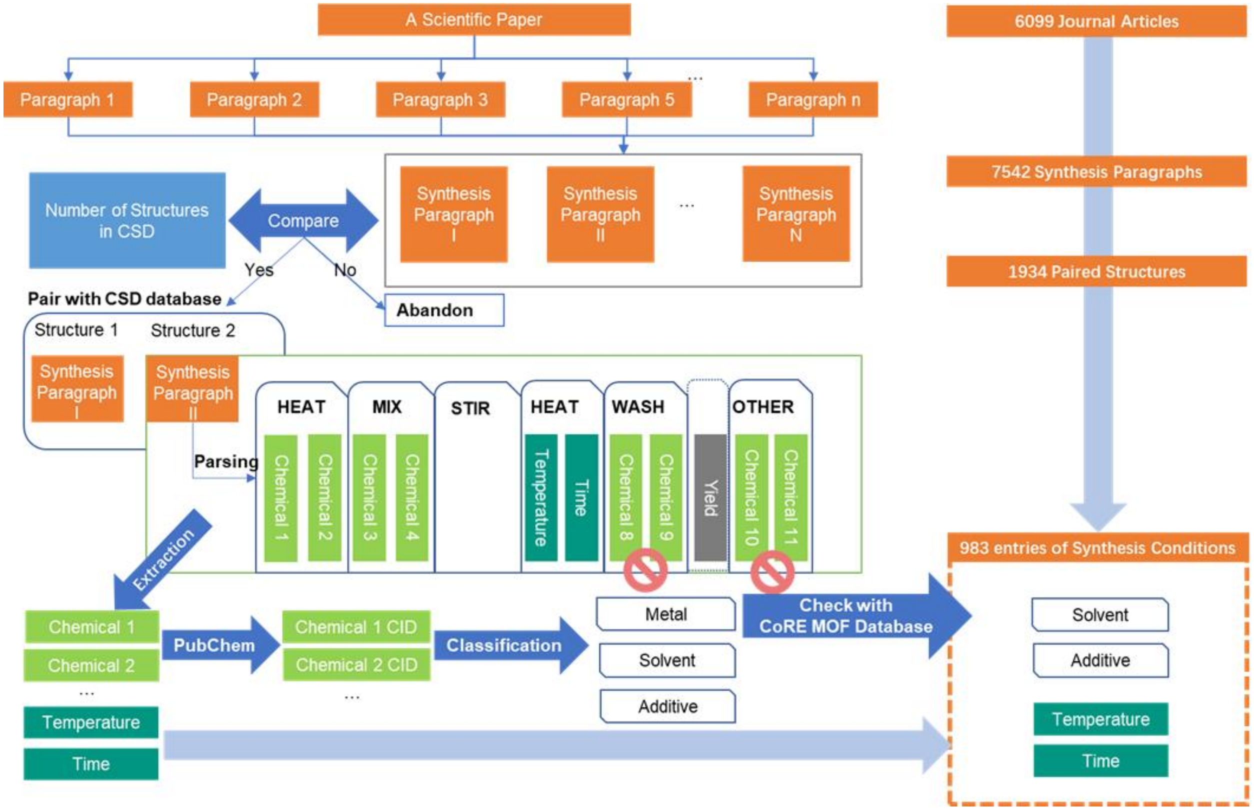

Figure S3. Data mining workflow from MOF literature. *Initially, each paper is subdivided into the paragraphs. Afterwards, the paragraph containing synthesis information was marked as "Synthesis Paragraph" and the corresponding MOF structure was assigned. The synthesis information was then extracted and transformed into PubChem CID.*[2]

2.2. Decision tree algorithm for paragraph classification.

We used a string search method to select synthesis paragraphs from publications. The literature content was initially divided into paragraphs by ChemDataExtractor software[3]. In the next step, all the paragraphs were classified as "Synthesis Paragraph" or "Irrelevant Paragraph" by a developed decision tree algorithm based on a keyword list selected from 100 MOF synthesis papers. (**Figure S4**).

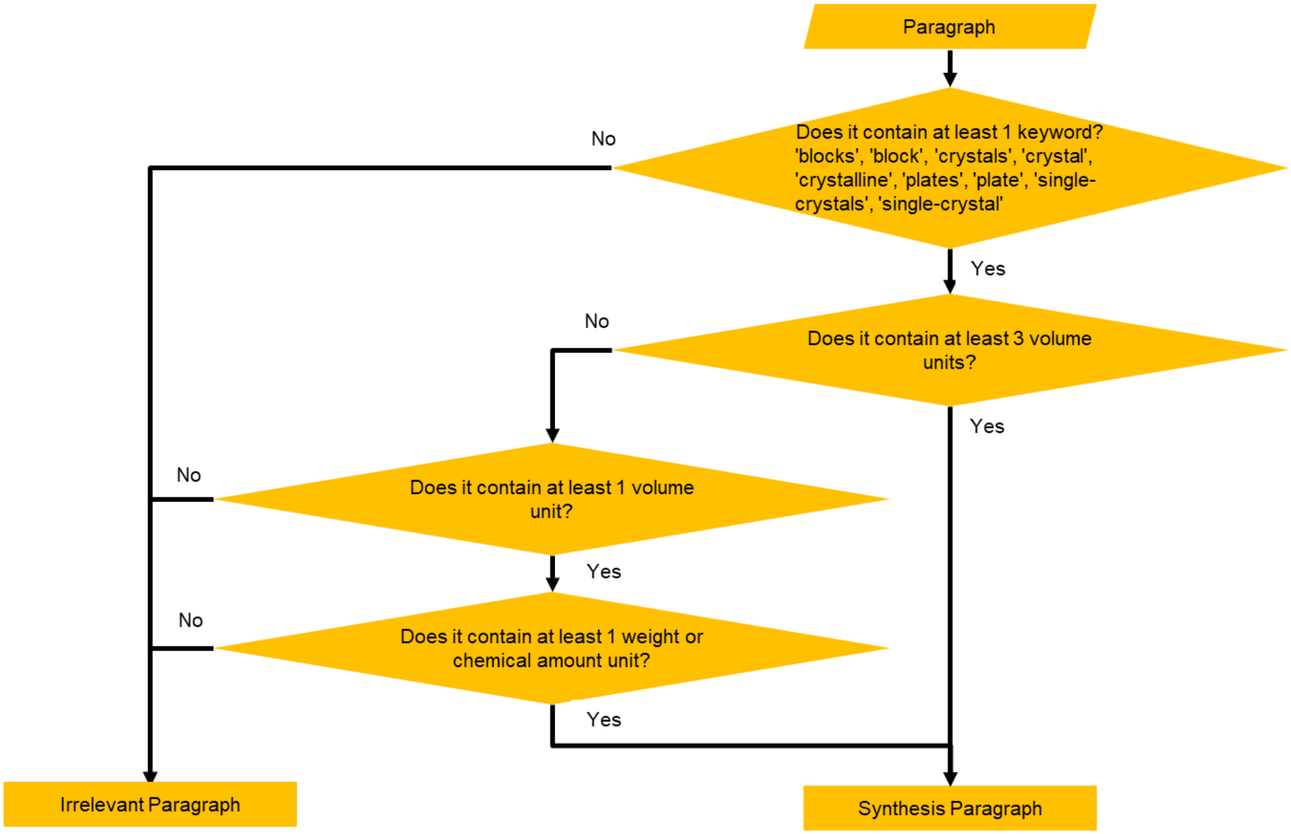

Figure S4. *Decision tree algorithm for paragraph classification.*

2.3. MOF synthesis conditions extraction

To analyze the synthesis paragraph and identify information on chemical entities, and corresponding experimental conditions, we applied the ChemicalTagger software[4] to understand each sentence grammatically and identify significant nouns inside the sentences. The significant nouns were then selected to determine the synthesis conditions (temperature, time) and chemical entities (chemical names and

quantity). Afterwards, the phrases inside the paragraph were annotated with different operation tags, including “Heat”, “Mix”, “Stir”, “Add” and “Wash” (**Figure S3**). In order to increase the accuracy of the ChemicalTagger for MOF literature extraction, we modified the input paragraph (**Table S1**).

Table S1. *Text modification of the input paragraph.*

**Original words (Content in quotation marks)**	**Modified words (Content in quotation marks)**
'silica gel plates'	'silica gel'
'building block'	'building linker'
'building blocks'	'building linkers'
'without stirring'	''
'distilled water'	'water'
'aqueous'	'water'
'°C/'	'°C for '
'· '	'·'
'is about'	'is '
'. The yield'	', the yield'
'methanolic'	'methanol'
' DMA '	' Dimethylacetamide '
' DMAc '	' Dimethylacetamide '
'pH≈'	'pH ≈ '
'pH='	'pH = '
' N ,N'	' N,N'
'·2.5 H2O'	'·2.5H2O'

When precursors, solvents and additives, as well as synthesis conditions were extracted, we transformed chemical names into corresponding CIDs in PubChem. After completing synthesis procedure extraction,

we compared the metal source from the automatically formed protocol to the one in the CoRE MOF database to eliminate mismatched conditions. The results of this fully automated data extraction were collected in the SynMOF-A database.

2.4. SynMOF databases

To evaluate the accuracy of the automatically extracted SynMOF-A database, we additionally extracted the synthesis conditions, manually creating SynMOF-M and SynMOF-ME databases (**Table S2**). The SynMOF-ME is an extended version of the SynMOF-M database, including information on the solvent ratio and metal counterion that currently cannot be extracted automatically using NLP.

Table S2. *Difference between the SynMOF databases.*

Database	Solvent	Additive	Temperature	Synthesis time	Metal Counterions	Solvent Ratio
SynMOF-A	✓	✓	✓	✓		
SynMOF-M	✓	✓	✓	✓		
SynMOF-ME	✓	✓	✓	✓	✓	✓

Comparison of the SynMOF-A with the SynMOF-M databases (**Table 3**) revealed that 928 paragraphs (94.4 %) were classified correctly. Among the 928 structures, 732 (78.9 %) structures are fully consistent with the manually extracted data with respect to temperature, time, solvent, and additive (**Table S3d**). Our data mining workflow showed good performance with respect to time and temperature extraction (97.7 %) (**Table S3b**). Since additive and solvent description is less standardized and the chemical components are more diverse and complex, consistency for additive and solvent extraction is slightly lower (81.3 %).

Table S3. *SynMOF-A database consistency with SynMOF-M database. a) Consistency of each component individually; combined consistency of b) time and temperature; c) additive and solvent; d) solvent and additive, as well as time and temperature.*

a)

	Time	Temperature	Solvent	Additive
Consistency	919 (99.0%)	910 (98.1%)	882 (95.0%)	792 (85.3%)

b)

	Temperature Consistency	Temperature Inconsistency
Time Consistency	906 (97.6%)	13 (1.4%)
Time Inconsistency	4 (0.4%)	5 (0.5%)

c)

	Additive Consistency	Additive Inconsistency
Solvent Consistency	753 (81.1%)	129 (13.9%)
Solvent Inconsistency	39 (4.2%)	7 (0.8%)

d)

	Time and Temperature Consistency	Time and Temperature Inconsistency
Solvent and Additive Consistency	732 (78.9%)	21 (2.3%)
Solvent and Additive Inconsistency	174 (18.7%)	1 (0.1%)

2.5. SynMOF database visualization

Two different featurization methods were used for ML models: RACs featurization and fingerprint based featurization (See 3.1 and 3.2 in SI). For RACs featurization, 671 MOFs of 983 MOFs were taken from the SynMOF. For fingerprint based featurization, 550 MOFs of 983 MOFs were taken from the SynMOF. To visualize the content of the SynMOF database used in the ML models, the following 3D bar diagrams are provided:

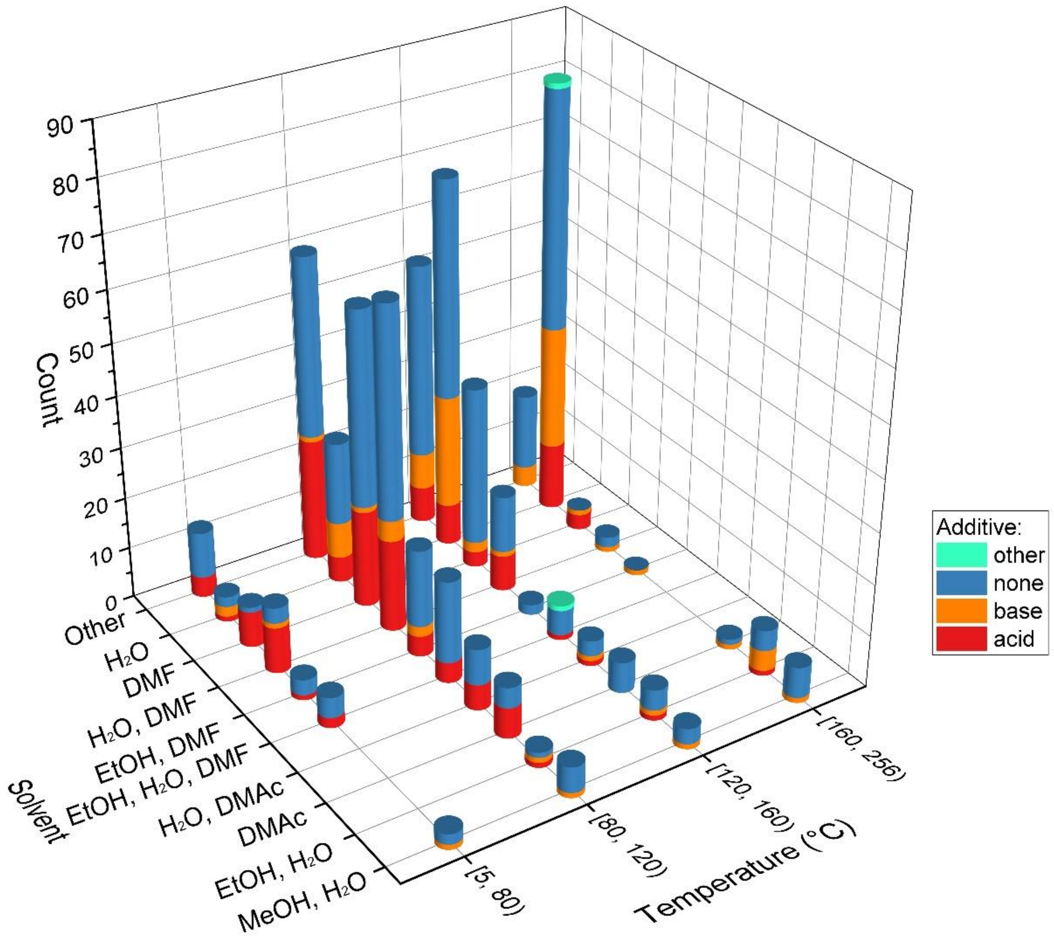

Figure S5. *Composition of the 671 entries in SynMOF-A database for ML model showing additive type as a color code vs. temperature and solvents used in MOF synthesis procedures.*

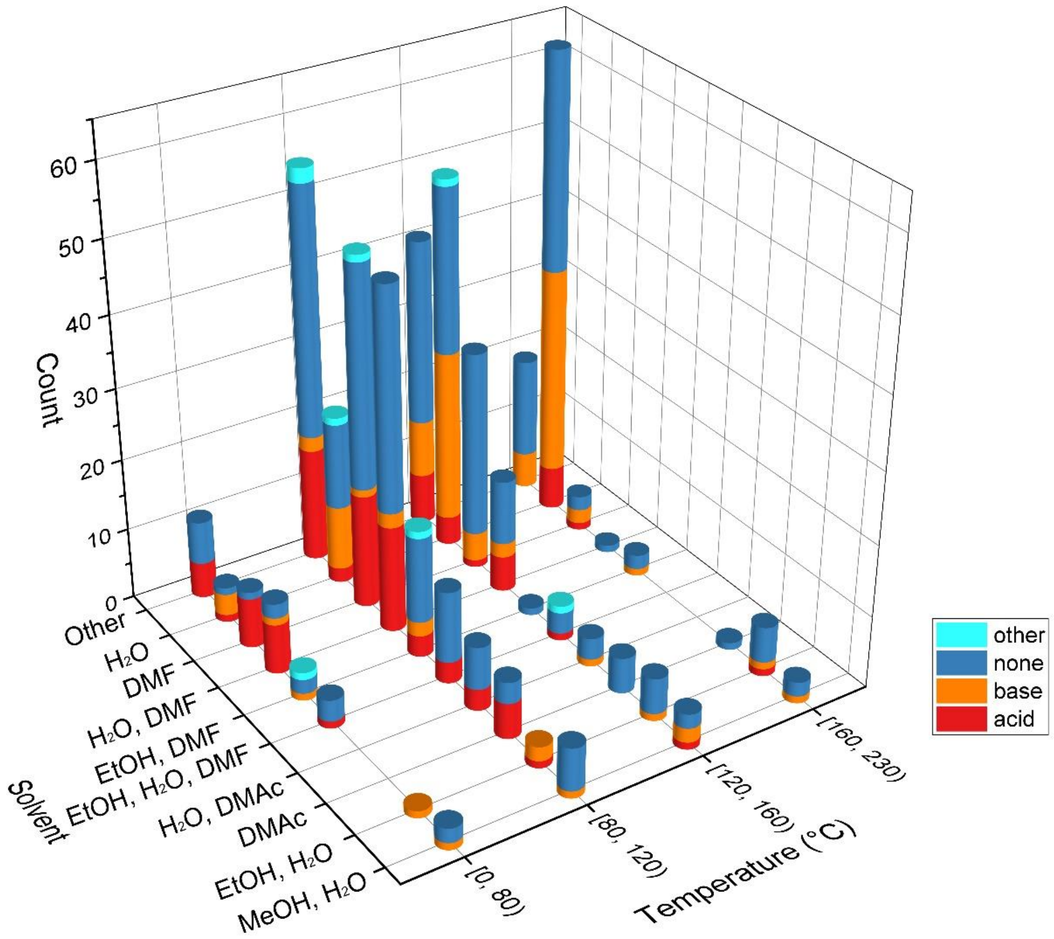

Figure S6. *Composition of the 550 entries in SynMOF-A database for ML model showing additive type as a color code vs. temperature and solvents used in MOF synthesis procedures.*

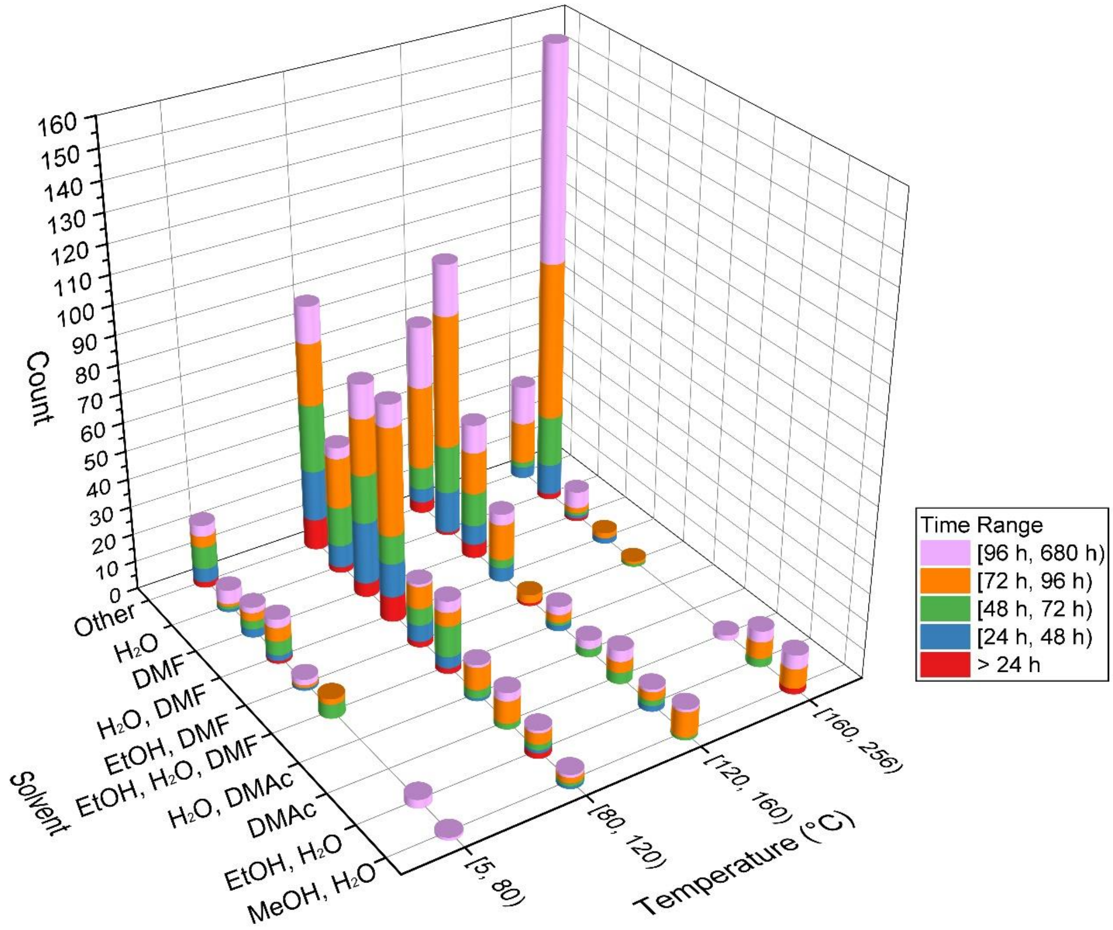

Figure S7. *Composition of the 983 entries in SynMOF-A database for ML model showing distribution of oxidation state as a color codes vs. temperature and solvents used in the corresponding MOF synthesis procedures.*

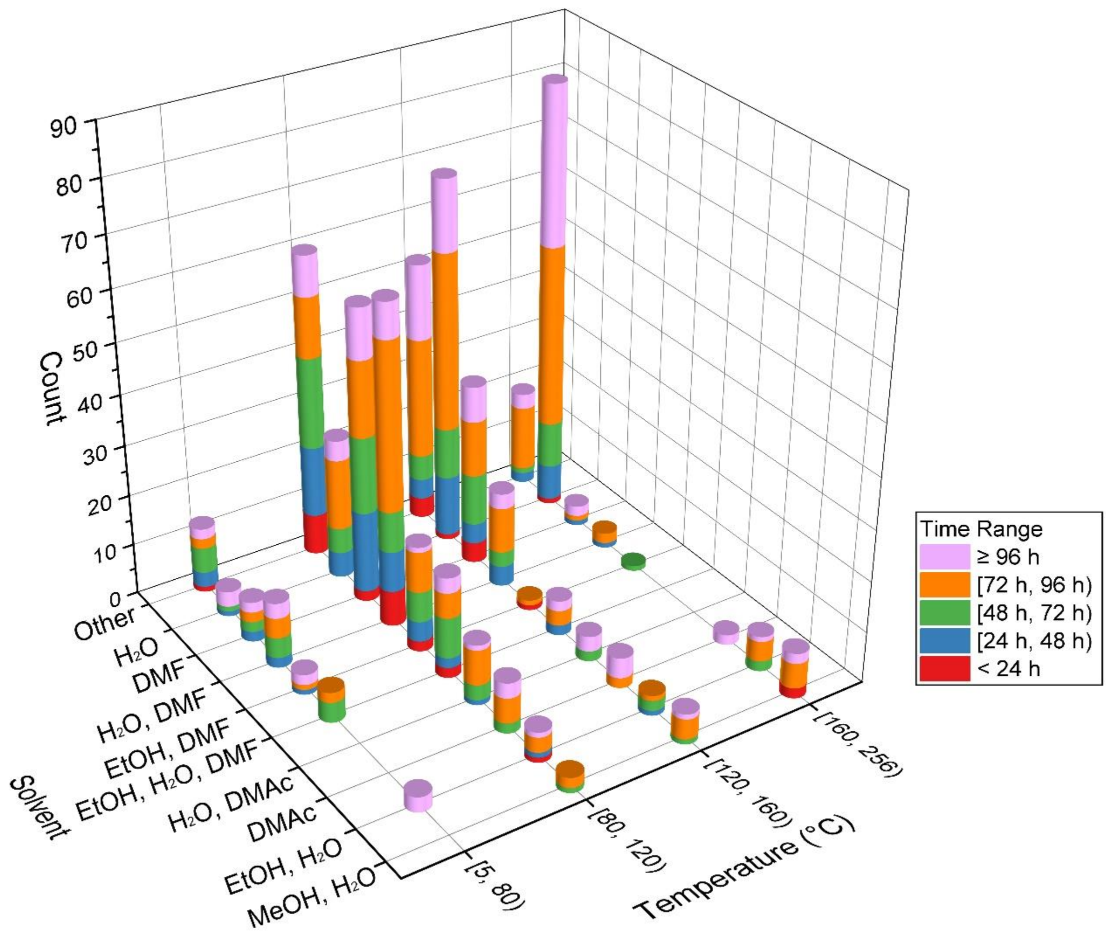

Figure S8. *Composition of the 671 entries in SynMOF-A database for ML model showing distribution of oxidation state as a color codes vs. temperature and solvents used in the corresponding MOF synthesis procedures.*

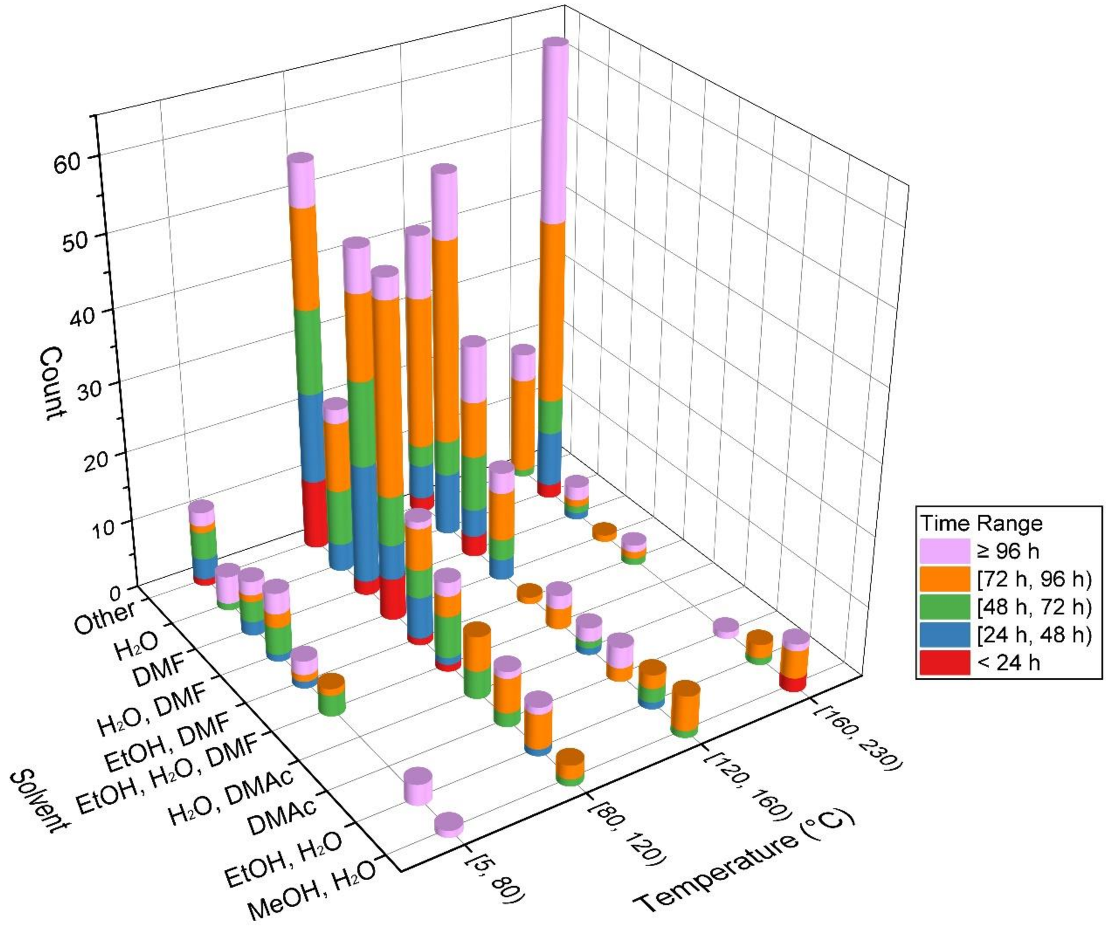

Figure S9. *Composition of the 550 entries in SynMOF-A database for ML model showing distribution of oxidation state as a color codes vs. temperature and solvents used in the corresponding MOF synthesis procedures.*

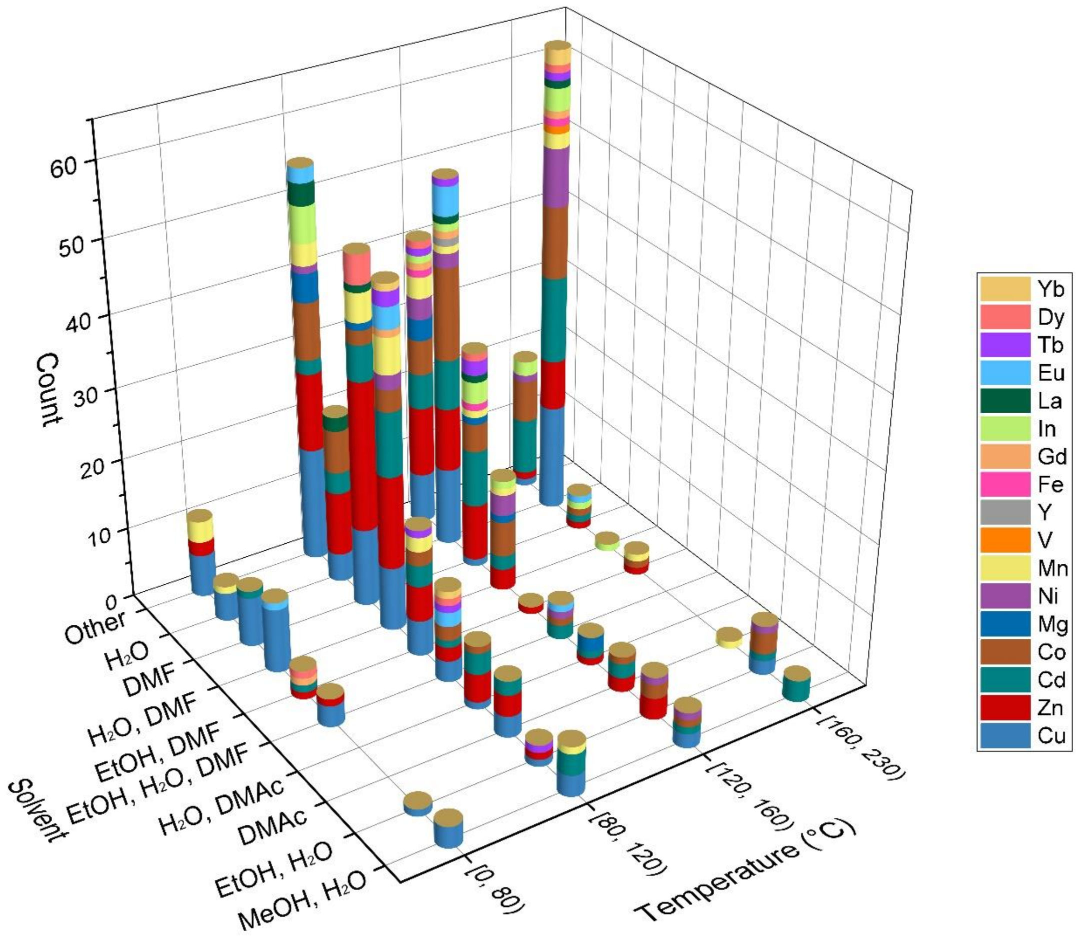

Figure S10. *Composition of the 550 entries in SynMOF-A database for ML model showing metal elements as a color code vs. temperatures and solvents used in MOF synthesis procedures.*

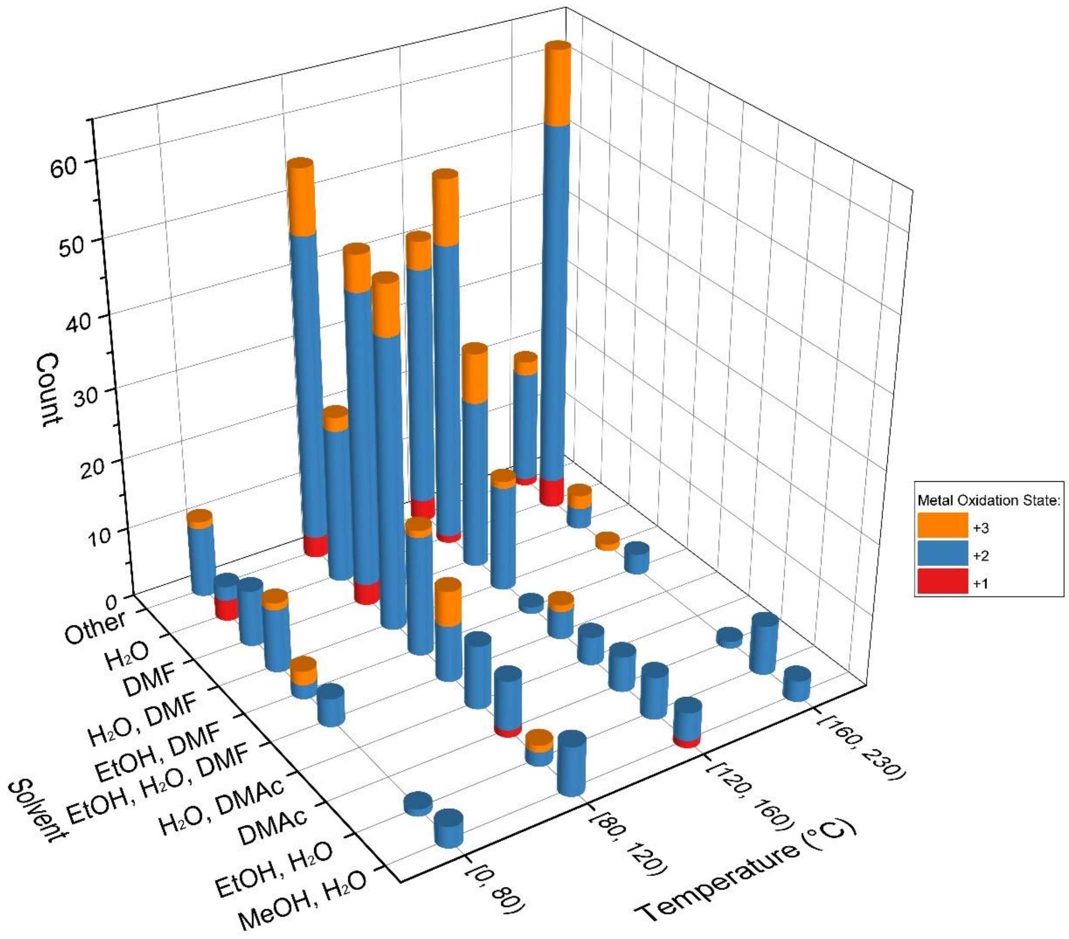

Figure S11. *Composition of the 550 entries in SynMOF-A database for ML model showing oxidation states as a color code vs. temperature and solvents used in MOF synthesis procedures.*

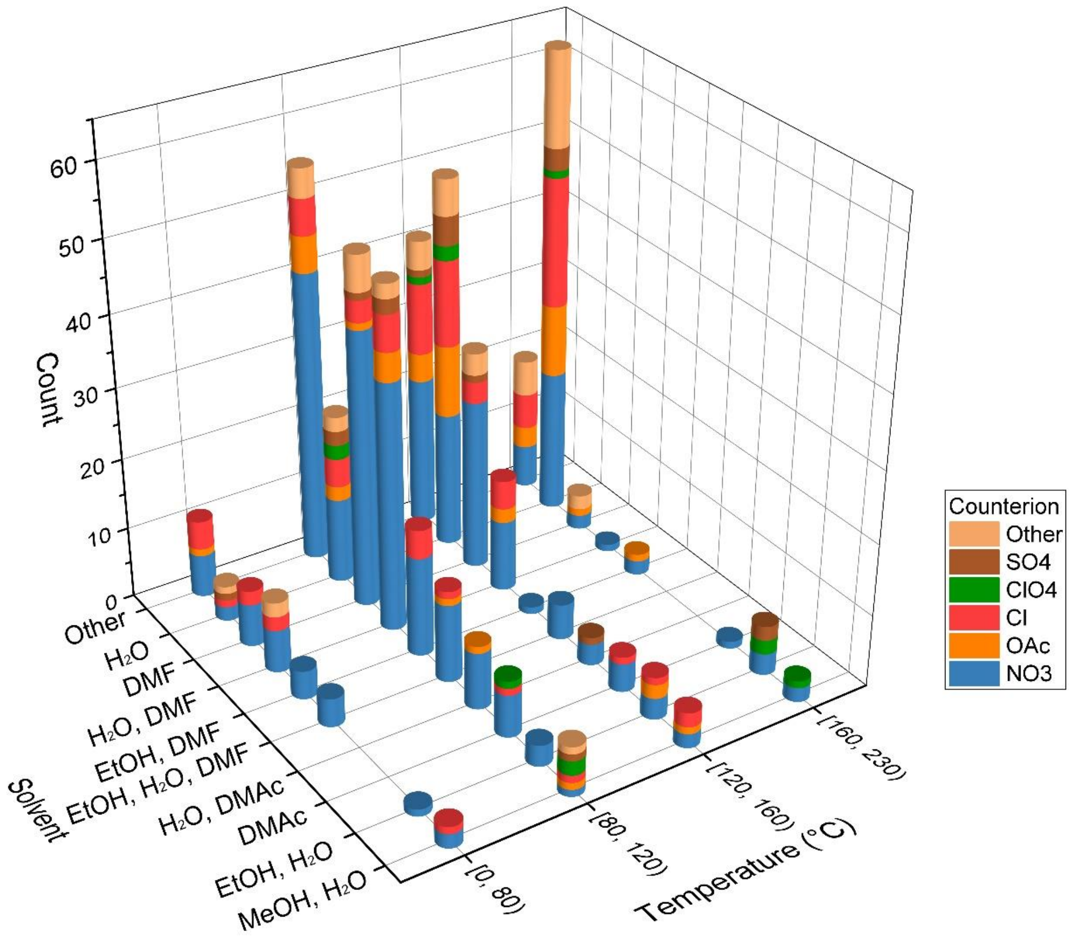

Figure S12. *Composition of the 550 entries in SynMOF-M database for ML model database showing counterions in the metal precursors as a color code vs. temperature and solvents used in MOF synthesis procedures.*

3. Details on the machine learning models

3.1. Fingerprint-based featurization

We developed an in-house code to extract the linkers from the CIF of the MOFs. The MOF unit cell was periodically extended in all three dimensions to generate a super cell. From these super cells, all organic fragments were extracted. The extraction process was based on the identification of chemical bonds between two atoms given their positions and distances, as well as the position of metal nodes. The fragments were filtered to remove solvents, additives and other molecules, and to finally identify and extract the linkers.

The RDKit library was further used to evaluate the molecular RDKit-fingerprints of the extracted linkers. A bit-vector of size 512 was used as the molecular fingerprint in this case. It's worth noting that we tried with the different size of the bit vector and chose the one that gave the best performing ML models. The metal nodes of the MOFs were represented by their full electronic configuration. Specifically, the electronic configuration of “Cu” was written as $1s^2$, $2s^2$, $3s^2$, $4s^1$, $5s^0$, $6s^0$, $2p^6$ $3p^6$, $4p^0$, $5p^0$, $3d^{10}$, $4d^0$, $5d^0$, $4f^0$ and the electron occupation of all these orbitals was used as input to the ML model. Thus, the input vector of the ML model reads as [2, 2, 2, 1, 0, 0, 6, 6, 0, 0, 10, 0, 0, 0]. We used the oxiMachin[6] model to assign the oxidation state of each metal cation inside the MOF structure from the corresponding CIF. The molecular fingerprint of the linkers, the full electronic configuration of the metal node and the oxidation state of the metal nodes are concatenated together to form the input of the ML model. For the MOFs with two different linkers A and B, two data points are created by changing the order of concatenation of linker fingerprints. In detail, if the fingerprint of the linker A is fp_A and B is fp_B, then in one data point the overall fingerprint is constructed by concatenating the fingerprints as [fp_A, fp_B], while another data point was created by concatenating them as [fp_B, fp_A]. This was done to ensure that the ML models do not differentiate the input based on the order of concatenation of the linker fingerprints. While training the ML models, we ensured that both of these two data points remain either in the training or test dataset. In the case of MOFs with only one linker, only one data point was prepared by concatenating the same fingerprint twice as [fp_A, fp_A].

3.2. Features from Kulik and co-workers

Kulik and co-workers developed feature vectors for MOFs by combining features of pore geometries and chemical components (*i.e.*, metal nodes, ligands, and functional groups) of the MOFs. The pore geometry was described as a simple geometric descriptor including pore size and volume. To describe the MOF chemistry, revised autocorrelations RACs[5] to include descriptors for all domains of a MOF material were used, namely metal chemistry, linker chemistry, and functional groups. For all our calculations, we directly used these features (CoRE MOF-2019 database) provided as the Supplementary Information of their publication.

3.3. Random forest (RF) regression models

RF regression models were implemented using the scikit learn library (sklearn.ensemble.RandomForestRegressor). The number of trees in the RF model was kept at 100. The depth of the trees was varied to find the best performing ML models for different regression tasks. For the best performing models, the depth of the trees was found out to be between 5 to 15. The other parameters were kept at default values. The entire dataset was split into different train-test sets using k-fold cross-validation. We generated 10 different train-test splits and trained 10 different ML models. The accuracy of the ML model predictions was quantified by calculating the MAE and correlation coefficient r^2 of the train and test dataset. To estimate the overall MAE, the mean and standard deviation was calculated from the 10 MAE values obtained from 10 different test-train splits as mentioned above.

3.4. Random forest (RF) classification models

RF classification models for additive prediction were implemented using the scikit learn library (sklearn.ensemble.RandomForestClassifier). The number of trees in the RF model was kept at 100 while maximum depth of the trees was fixed at 5. The dataset for the classification task was balanced using the

class_weight = balanced keyword. The accuracy of the trained classification models were calculated by evaluating the confusion matrix.

3.5. Neural Network (NN) regression models

The NN regression model was implemented using the TensorFlow.Keras library. The NN hyperparameter optimization was done using a random search over the hyperparameter space. The details of the parameter space scanned are as follows:

The number of layers of the NNs varied between 1 and 5. The number of neurons in the first hidden layer N_1 was randomly chosen between 10 to 1000. The numbers of neurons in the next hidden layers were chosen according to the relation $N_1 \cdot f^{k-1}$. Here, k is the hidden layer index, and f was chosen between 0.1 and 1. The learning rate was chosen between 10^{-1} to 10^{-4}. The L2 regularization scheme with regularization parameter between 10^{-1} to 10^{-6} was selected. A LeakyReLU activation function with parameter *alpha = 0.005* was chosen in all layers except the output layer where a linear activation function was used.

For each randomly chosen set of hyperparameter values, we generated 10 different train-test splits using a k-fold cross-validation and trained 10 different ML models. The accuracy of the ML model predictions was quantified by calculating the MAE and correlation coefficient r^2 of the train and test dataset. To estimate the overall MAE, the mean and standard deviation was calculated from the 10 MAE values obtained from 10 different test-train splits as mentioned above.

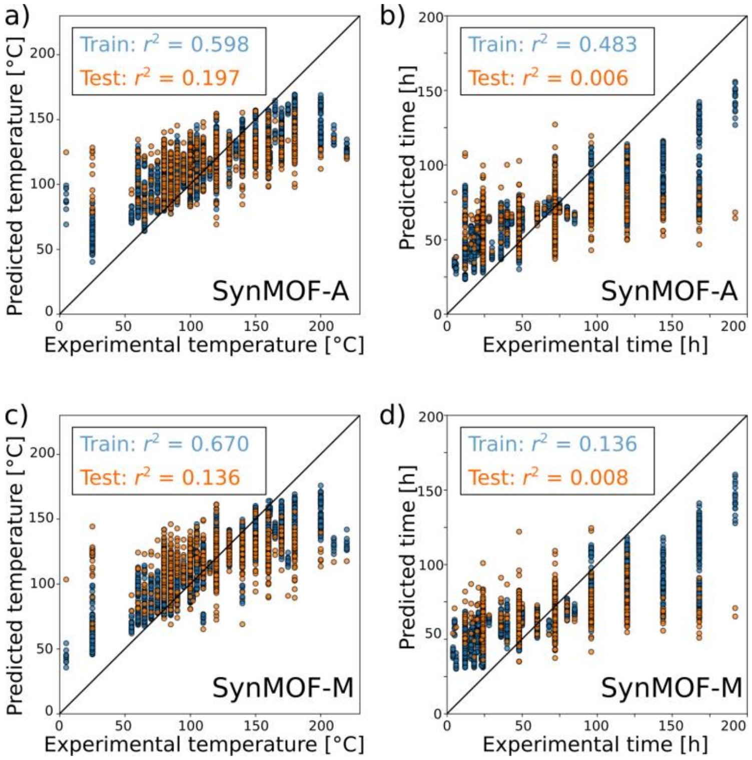

Figure S13. ML models based on fingerprint features. *a) and b) show synthesis temperature and time predictions using ML models trained on fingerprint features of the SynMOF-A database, while the models in c) and d) are trained on fingerprint features of the SynMOF-M database. We find that fingerprint-based features lead to slightly decreased performance of the ML models compared to the MOF features developed by Kulik and co-workers.*

3.6. Solvent prediction

To predict the solvents used in the synthesis, we use a nearest-neighbor search in solvent properties space. The algorithm for this search is described in-detail as follows. The solvents were represented by five relevant properties (water-octanol partition coefficient *logp*, number of hydrogen bond donors, number of hydrogen bond acceptors, and maximum absolute partial charge, boiling point). These five properties were standard-scaled and used as an output of the ML regression models. We denote the five properties for the solvent of MOF *j* as a vector p^{solvent}_i, and the corresponding ML prediction as p^{predict}_i. In property space, we calculated the distance of the predicted solvent properties from all the 31 actual solvents found in the database as follows

$$d^{\text{pred}}_{kj}=[\textstyle\sum_i (p^{\text{pred}}_{ji} - p^{\text{solvent}}_{ki})^2]^{0.5}$$

The distances were ordered and closest m (m was varied between 1 and 5) solvents are used as top-m predictions (see **Figure 3e**).

As a random baseline reference, we used three methods. Firstly, we performed the nearest neighbor search with randomly chosen solvent properties. In addition, we calculated the probability of finding the correct solvent by simply suggesting random solvents from the full list of 31 solvents or from the list of 6 most frequently occurring solvents in the database.

The procedure presented above only works for MOFs with synthesis conditions that only require a single solvent. However, many MOFs in the SynMOF database use more than one solvent (two or three). For the results presented in this study, we limited the solvent prediction to the subset of MOFs with only one solvent. However, in principle the algorithm presented above can also be extended to multiple solvents. It is possible to predict the main solvent as well as the second-solvent direction in solvent property space, and use both quantities to sample solvent mixtures. Due to a limited amount of currently available data, we plan to implement and evaluate this in the future.

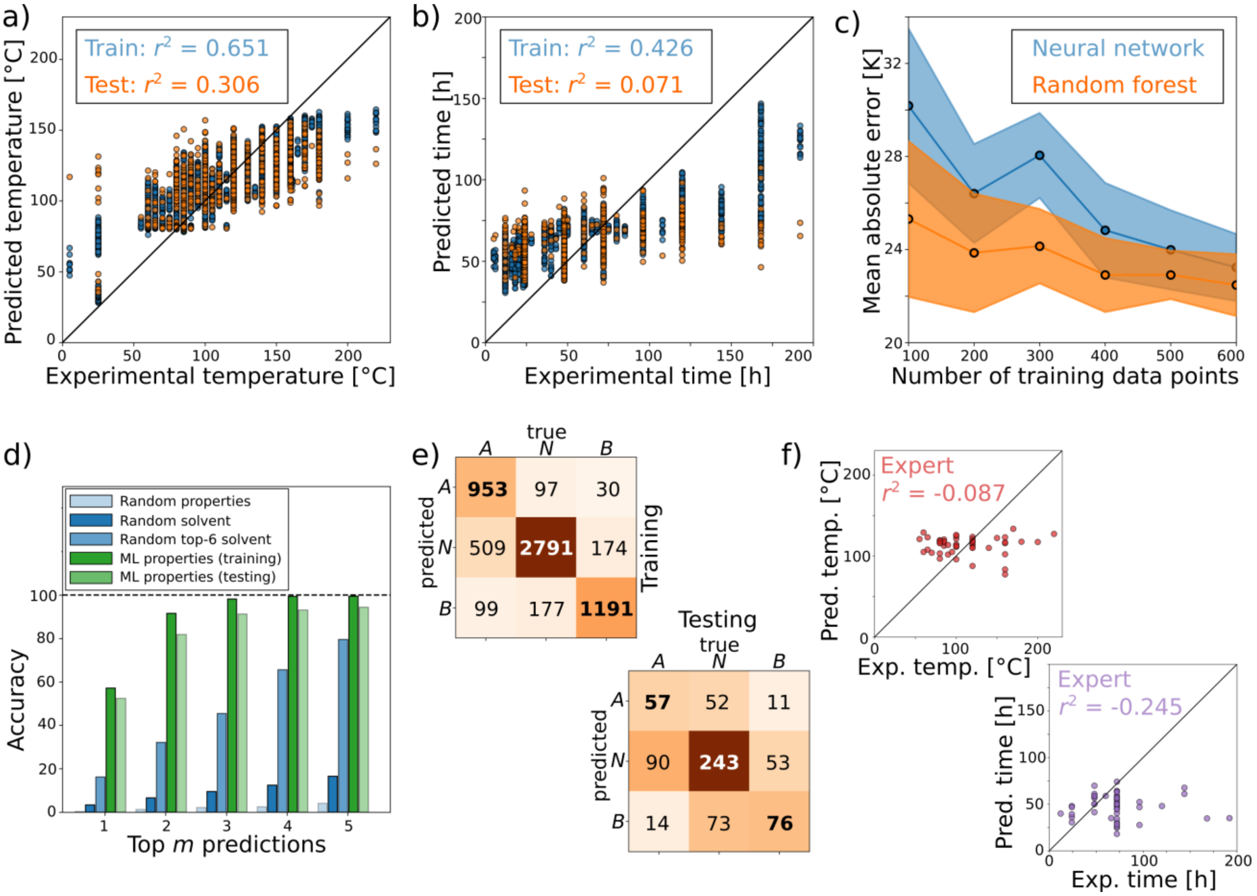

Figure S14. ML models trained on SynMOF-M database. *Panels a-f) correspond to panels b-g) of Figure 3 in the main manuscript. Here, all ML models are trained on the manually corrected SynMOF-**M** database while the main manuscript shows results on the automatically extracted data in the SynMOF-**A** database. Only minor changes compared to the SynMOF-A results are visible.*

3.7. Details on expert survey

We designed an expert survey to estimate the complexity of the MOF synthesis prediction task. In this survey, eleven MOF synthesis experts from MOF research groups (Dr. Christian Diercks (Scripps Research Institute), Dr. Julien Reboul (Sorbonne Université), Dr. Roberto Fernández de Luis (BCMaterials), Dr. João Marreiros (KU Leuven), Dr. Stéphane Diring (Nantes University), Dr. Akira Hinokimoto, Dr. Eli Sanchez Gonzalez, Dr. Javier Troyano (Kyoto University) and Dr. Romy Ettlinger (University of Augsburg), as well as the co-authors of this study, Dr. Jacopo Andreo and Prof. Dr. Stefan Wuttke (BCMaterials) were asked to predict the synthesis conditions for a set of 50 MOF structures, without the help of literature sources (see SI template). The survey was built on a jupyter notebook made accessible

using Google Colab (**Figure S12a, see SI survey**). As an input, the experts received detailed information on molecular structure of the linker, metal type and oxidation state, as well as the 3D structure of the target MOF. **Figure S12b** shows the information provided to the MOF synthesis experts for one of the 50 MOFs in the quiz. A full overview is provided as an additional supplementary file. The experts were then asked to predict the time, temperature, and concentrations of metal and linker components as free input fields. In addition, the experts were asked to select solvent(s), additive, and counter ion of the metal source from a drop-down menu containing all solvents and counter ions contained in the SynMOF database and for additives the choices “acid”, “base” or “none”.

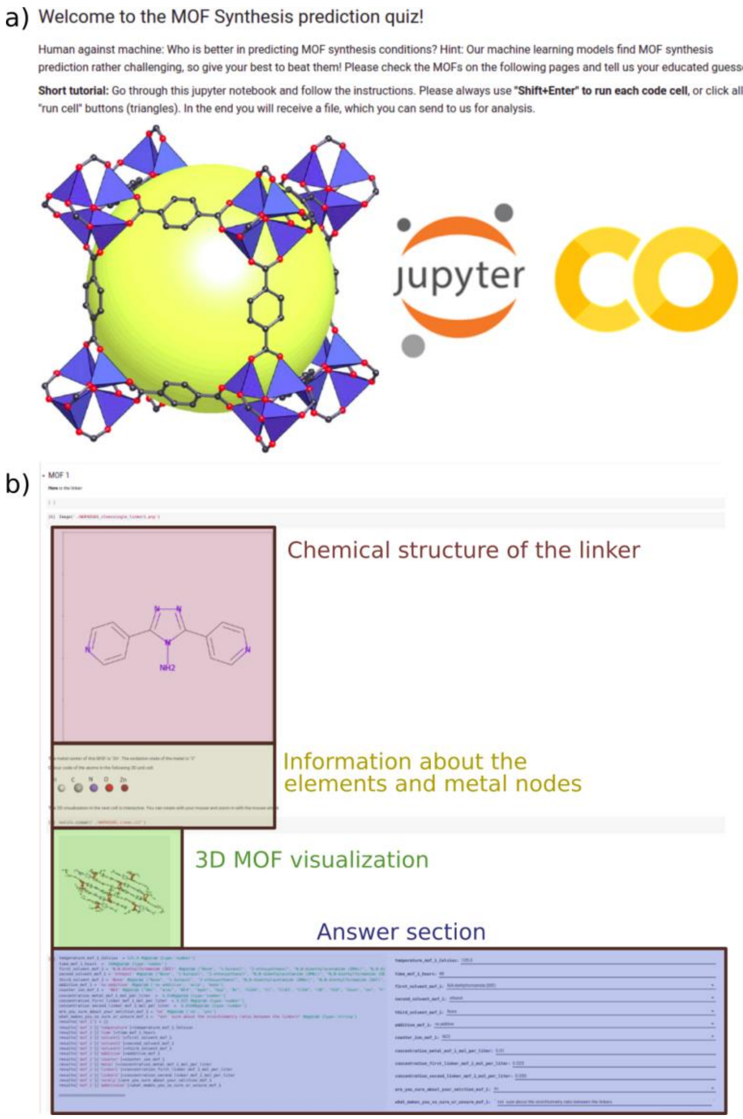

*Figure S15. **Information provided to the MOF synthesis experts.** a) Overview of the quiz built on jupyter and Google Colab. b) Information provided to the experts. A full version of the quiz is provided as an additional supplementary file.*

3.8. Demo for the web-tool

To use the web-tool for synthesis prediction, please upload a CIF of the desired MOF structure under https://mof-synthesis.aimat.science/upload/
As a demo we provide a cif of MOF-5 for download on the website.
Once the cif is uploaded, the predicted temperature, time, solvent, and the used of additives are printed below. The screenshot in Figure S16 shows the predicted conditions for MOF-5

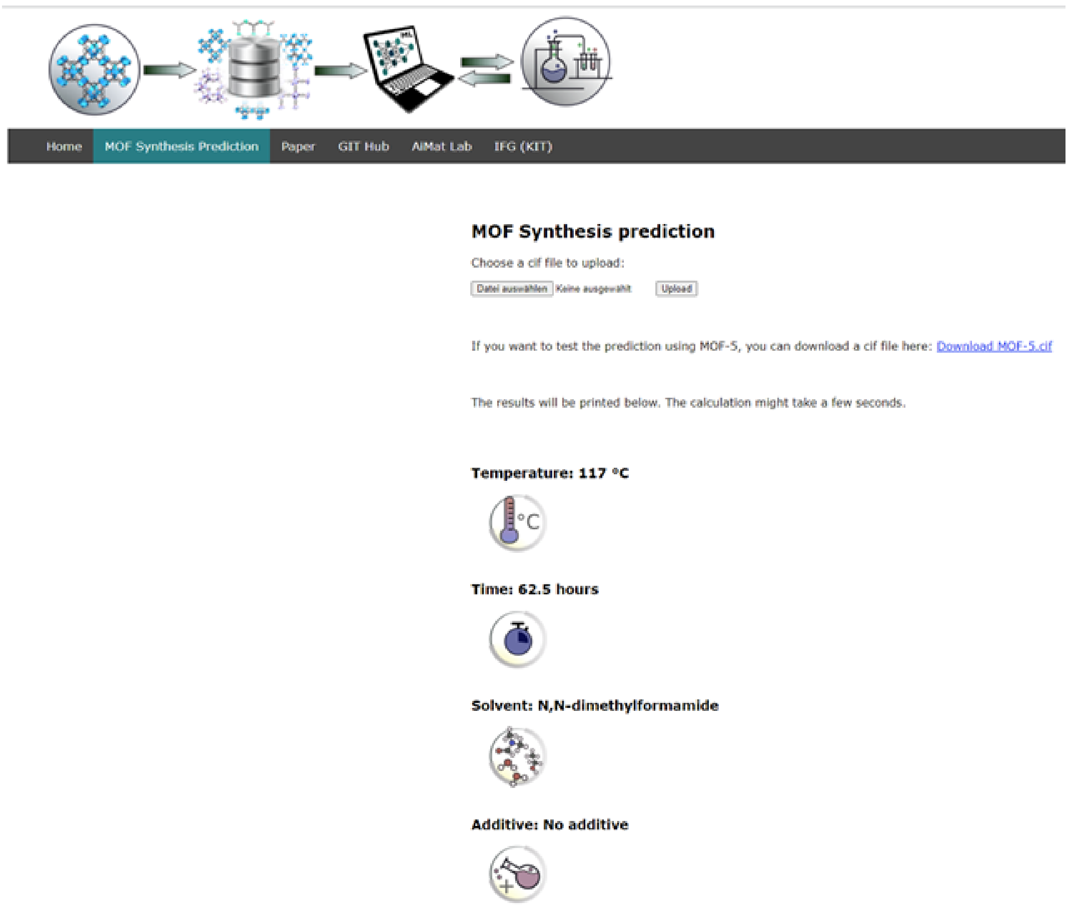

***Figure S15. MOF synthesis prediction tool.** Synthesis prediction web-tool, with conditions predicted for MOF-5.*

References

1. Groom, C. R., Bruno, I. J., Lightfoot, M. P. & Ward, S. C. The Cambridge Structural Database. *Acta Crystallogr., Sect. B: Struct. Sci., Cryst. Eng. Mater.* **72**, 171–179 (2016).

2. Kim, S. *et al.* PubChem in 2021: new data content and improved web interfaces. *Nucleic Acids Res.* 49, D1388–D1395 (2021).
3. Swain, M. C. & Cole, J. M. ChemDataExtractor: A Toolkit for Automated Extraction of Chemical Information from the Scientific Literature. *J. Chem. Inf. Model.* 56, 1894–1904 (2016).
4. Hawizy, L., Jessop, D. M., Adams, N. & Murray-Rust, P. ChemicalTagger: A tool for semantic text-mining in chemistry. *J. Cheminf.* 3, 17 (2011).
5. Moosavi, S. M. *et al.* Understanding the diversity of the metal-organic framework ecosystem. *Nat. Commun.* 11, 4068 (2020).
6. Jablonka, K. M., Ongari, D., Moosavi, S. M. & Smit, B. Using collective knowledge to assign oxidation states of metal cations in metal–organic frameworks. *Nat. Chem.* 1–7 (2021).
